# Real-World Prescribing Patterns and Treatment Continuation of Amitriptyline Monotherapy and Aripiprazole Augmentation for Medically Unexplained Oral Symptoms/Syndromes in Japan

**DOI:** 10.3390/ph18091282

**Published:** 2025-08-27

**Authors:** Chizuko Maeda, Takayuki Suga, Takahiko Nagamine, Akira Toyofuku

**Affiliations:** 1 Department of Psychosomatic Dentistry, Graduate School of Medical and Dental Sciences, Institute of Science Tokyo, Bunkyo 113-8510, Japan; mtccs.ompm@tmd.ac.jp (C.M.); tnagamine@outlook.com (T.N.); toyoompm@tmd.ac.jp (A.T.); 2 Department of Psychiatric Internal Medicine, Sunlight Brain Research Center, Hofu 747-0066, Japan

**Keywords:** medically unexplained oral symptoms/syndromes, real-world evidence study, pharmaceutical therapy

## Abstract

**Background:** Medically unexplained oral symptoms/syndromes (MUOS), such as Burning Mouth Syndrome and Persistent Idiopathic Facial Pain, present significant management challenges due to the lack of standardized treatments and high-level evidence. While pharmacotherapy is often employed, real-world data on treatment adherence—a pragmatic proxy for effectiveness and tolerability—remain sparse, especially in Japan. This study aimed to describe the real-world prescribing patterns of antidepressants and dopamine receptor partial agonists (DPAs) for MUOS and retrospectively investigate their association with treatment continuation. **Methods:** This retrospective observational study analyzed data from patients initiating pharmacotherapy for MUOS at a specialized clinic in Japan (April 2021–March 2023). We used Cox proportional hazards models to evaluate treatment continuation for amitriptyline monotherapy and antidepressant–aripiprazole adjunctive therapy. The primary outcome was the time to discontinuation. Dosage effects were modeled using B-splines to capture nonlinearity. **Results:** Among 702 MUOS patients who started pharmacotherapy, 493 received amitriptyline as the first prescription, and 108 received aripiprazole as an adjunctive therapy. For amitriptyline monotherapy, a nonlinear relationship was observed between dosage and discontinuation risk, with a relatively lower hazard around 25 mg/day across age groups. In the antidepressant–aripiprazole adjunctive group, the overall hazard ratio for discontinuation was higher (HR = 4.75, *p* < 0.0005) compared to non-adjunctive therapy, likely due to indication bias reflecting more treatment-resistant cases. However, within the aripiprazole adjunctive group, a U-shaped relationship was identified between maximum aripiprazole dosage and discontinuation risk, with the lowest hazard (HR ≈ 0.30) observed at approximately 1.7–1.8 mg/day. Mild side effects such as drowsiness, dry mouth, constipation, tremor, insomnia, and weight gain were noted, but no severe adverse events occurred. **Conclusions:** This real-world data analysis suggests specific dosage ranges (amitriptyline ≈ 25 mg/day; aripiprazole augmentation ≈ 1.7–1.8 mg/day) are associated with longer treatment continuation in MUOS patients. Treatment continuation reflects a crucial balance between symptom relief and tolerability, essential for managing these chronic conditions. It is critical to emphasize that these findings are descriptive and observational, derived from a specialized setting, and do not constitute prescriptive recommendations. They highlight the importance of individualized dosing. Definitive evidence-based strategies require validation through prospective randomized controlled trials.

## 1. Introduction

Medically unexplained oral symptoms/syndromes (MUOS) is a conceptual term for conditions in which patients report intraoral pain, burning sensation, numbness, or discomfort, yet no obvious physical abnormalities are identified. It has been suggested that multiple factors, such as psychosocial stress and functional changes in the central nervous system, may be involved in the onset of MUOS [1]. Previously, intraoral unpleasant sensations were strongly attributed to dental pathologies, but, in MUOS, even with detailed examinations, no organic abnormalities are confirmed, and linking the findings to appropriate care can be difficult. As a result, patients often continue to suffer for extended periods without sufficient explanation or treatment. Another significant concern is that, in the process of searching for the cause of these symptoms, repeated excessive dental interventions may be performed [2,3].

Representative conditions encompassed by MUOS include Burning Mouth Syndrome (BMS), Persistent Idiopathic Facial Pain (PIFP) (including Atypical Odontalgia: AO), Phantom Bite Syndrome (PBS), and oral cenesthopathy (Table 1). Notably, a recent conceptual trend advocates for reconsidering BMS and oral cenesthopathy collectively under the historical classification of oral dysesthesia (OD) (a term defined as abnormal unpleasant sensations in the oral cavity occurring without a clear identifiable physical cause) [4,5]. For example, BMS refers to cases where chronic pain or a burning feeling on the tongue is not explained by blood tests or imaging. Typical complaints include “a burning sensation on the tongue” or “a persistent tingling feeling.” Although this condition is frequently noted in women after menopause, it can be difficult to pinpoint any specific organic cause, and many patients have been reported to visit multiple dental offices [6]. Such MUOS conditions are believed to involve multifaceted factors, including psychological and social elements and changes in the central nervous system. Consequently, a single management method often proves challenging [7,8]. These entities share several hallmark clinical characteristics. First, patients typically experience persistent intra-oral dysesthesia—most often burning, tingling, or poorly localized pain—that is disproportionate to any detectable mucosal pathology [9,10]. Second, the disorders exhibit a female predominance with peak onset in the peri- to post-menopausal period [9,11,12,13]. Third, symptoms frequently co-exist with affective comorbidities such as anxiety, depression, and heightened somatosensory amplification, and are often exacerbated by psychosocial stressors and repeated unsuccessful dental interventions [12,14]. Collectively, these features justify grouping them under the umbrella of medically unexplained oral symptoms and highlight the need for integrated biopsychosocial treatment strategies [15].

In this study, we use the term MUOS as a clinical umbrella that is familiar to dentists; however, we also align our diagnostic entities with international classifications. In the International Classification of Diseases, 11th Revision (ICD-11), BMS is a distinct entity (DA0F.0), defined by chronic intraoral burning or dysesthetic pain without visible lesions and with associated distress or interference (pain on ≥50% of days for >3 months) [16]. In contrast, PIFP is represented within the cranial neuropathies/facial pains section and maps to atypical facial pain (8B82.1). From a psychiatric nosology, symptom constellations seen in MUOS may also meet criteria for somatic symptom-related conditions, namely Bodily Distress Disorder (BDD) in ICD-11 and Somatic Symptom Disorder (SSD) in the Diagnostic and Statistical Manual of Mental Disorders, Fifth Edition, Text Revision (DSM-5-TR), when disproportionate thoughts/feelings/behaviors about symptoms are prominent [17]. Thus, while dentistry benefits from retaining disorder-specific labels (BMS, PIFP, oral cenesthopathy, and Phantom Bite Syndrome/occlusal dysesthesia), we explicitly acknowledge their trans-diagnostic placement within BDD/SSD when appropriate.

Various medications, including tricyclic antidepressants (TCA), serotonin–noradrenaline reuptake inhibitors (SNRI), selective serotonin reuptake inhibitors (SSRI), benzodiazepines, and antipsychotics, may be used in drug therapy for MUOS. However, there are limited reports on real-world prescribing patterns in Japan, especially with a focus on treatment continuity [11,12,18,19].

In recent years, research suggests that pharmacotherapy centered on antidepressants may be beneficial for MUOS symptoms [20]. TCA has been extensively used as a treatment for MUOS and is reported to inhibit neuropathic pain and increase the pain threshold [11,12,19,21]. However, because MUOS patients display considerable variation in age, past medical history, and general health, it is necessary to judge “what dosage and duration are appropriate” carefully. In particular, TCA has strong anticholinergic effects that frequently cause side effects such as dry mouth, constipation, and dizziness, requiring especially careful dosage planning in older adults and patients with comorbid conditions [21]. At present, there are few large-scale studies or guidelines that have examined the dose–response relationship of antidepressants specifically for oral symptoms, and ‘how much the dose should actually be increased’ is currently determined through trial and error in clinical practice.

Meanwhile, in recent years, augmentation therapy that combines antidepressants with dopamine receptor partial agonists (DPAs) has also drawn attention [22]. DPAs modulate the dopaminergic and serotonergic systems and have shown benefits and safety in treatment-resistant depression [22,23,24,25]. Though evidence on MUOS is still limited, there are reports that adding DPAs to antidepressant monotherapy—which showed minimal change in symptoms—improved pain or discomfort [11,18,19,26]. By regulating dopamine and serotonin pathways, DPAs may correct cognitive or emotional imbalance, and potential neuroprotective and anti-inflammatory functions have been proposed, suggesting a multifaceted approach to MUOS [27,28,29].

Because there are no standardized regimens in real-world data, treatment dropout is an important indicator that comprehensively reflects changes over time in symptoms and tolerability. In this study, the primary outcomes were the duration of treatment continuation and the hazard ratio of treatment discontinuation. Additionally, it is crucial to understand MUOS as a “Complex Adaptive System (CAS),” which refers to a dynamic network where multiple components (e.g., biological, psychological, social factors) interact and self-organize, leading to emergent behaviors (like chronic pain) that cannot be fully explained by analyzing the individual components alone [30]. Given these characteristics, it is advisable to adopt multifaceted outcome measures rather than relying on a single indicator. Although “Full Functional Recovery (FFR)” has been proposed as a comprehensive goal in BMS, it may not be feasible to apply FFR universally to all MUOS cases when taking a CAS perspective [31]. The treatment continuation rate used in this study is considered a pragmatic measure that reflects multiple elements—including symptom improvement, side effects, and patient tolerance—in an integrated manner.

The purpose of this investigation is to describe the prescribing patterns of medications used to address MUOS in our department and to conduct a retrospective exploration focusing on the use patterns and treatment continuity of antidepressants and DPAs, which were frequently prescribed. This may offer descriptive insights into real-world prescribing for MUOS and potential factors related to treatment adherence.

## 2. Results

### 2.1. Association Between Amitriptyline Dosage and Treatment Continuation Among Patients Receiving Amitriptyline

**Figure 1:**
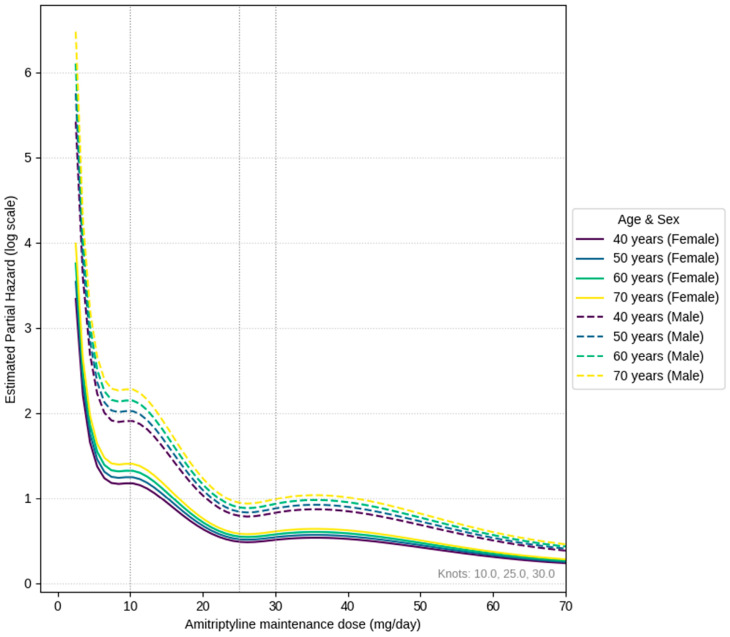
Estimated curve of hazard ratios for treatment discontinuation by amitriptyline dosage, stratified by age and sex. The graph shows age- and sex-specific hazard ratios derived from a Cox proportional hazards model, adjusted for covariates. The x-axis represents the amitriptyline maintenance dose, and the y-axis (log scale) represents the estimated partial hazard associated specifically with the dose, after accounting for the effect of age and sex. This illustrates the nonlinear relationship between dose and discontinuation risk. Note: The sample size for male patients is relatively small, requiring cautious interpretation of the corresponding curves.

Table 2 shows the background characteristics of the patients (age, sex distribution, average observation period, diagnoses in our department, etc.). Table 3 presents the findings from the Cox proportional hazards model regarding amitriptyline discontinuation, and Figure 1 shows a spline curve illustrating the relationship between dosage and hazard ratio. The main side effects were drowsiness, dry mouth, and constipation; importantly, no severe adverse events such as falls or cognitive impairment were recorded.

### 2.2. Association of Maximum Aripiprazole Dosage with Treatment Continuation in Adjunctive Antidepressant–Aripiprazole Therapy

Table 4 summarizes the background characteristics of the adjunctive aripiprazole group versus the non-adjunctive group, and Table 5 reports the Cox proportional hazards model results. Compared with patients not receiving adjunctive aripiprazole, the risk of treatment interruption in the adjunctive group was HR = 4.75 (*p* < 0.0005). However, because MUOS severity indicators (e.g., pain VAS, illness duration, response to previous treatment) were not adjusted for in this model, residual confounding due to indication bias is highly probable. Consequently, it is not valid to conclude solely from these findings that adjunctive aripiprazole impairs treatment continuity.

**Figure 2:**
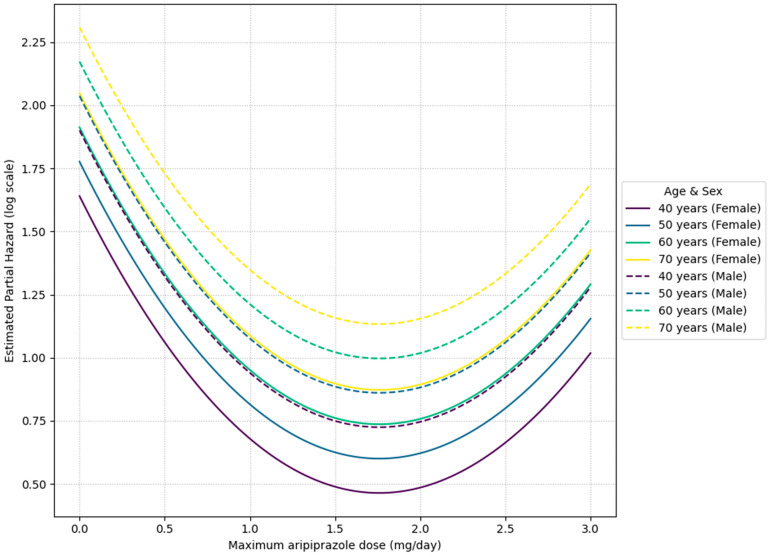
Estimated curve of hazard ratios for treatment discontinuation by maximum aripiprazole dosage used. The graph shows age- and sex-specific hazard ratios for patients in the adjunctive therapy group, derived from a Cox proportional hazards model. The x-axis represents the maximum aripiprazole dose, and the y-axis represents the estimated partial hazard. Note: The analysis revealed the minimum hazard range being around 1.7–1.8 mg/day overall. Because age-related differences were minimal, the Y-axis was plotted on a logarithmic scale. The sample size for male patients is small, requiring cautious interpretation.

Meanwhile, in the analysis restricted to the group receiving aripiprazole, a U-shaped relationship (Wald omnibus test *p* < 0.001) was suggested between maximum dosage and the risk of treatment discontinuation. Specifically, the hazard ratio was lowest (HR ≈ 0.30) around 1.7–1.8 mg/day. While an increased dosage up to around 1.8 mg/day corresponded to reduced risk, surpassing 1.8 mg/day was linked to a rise in risk again (Figure 2). Mild tremor, insomnia, and weight gain were observed as side effects in some cases, but none were severe, and no clinically significant changes in serum prolactin levels or other findings were detected.

## 3. Discussion

This study descriptively showed the prescription of antidepressants and DPAs to MUOS patients in real-world clinical practice. Among these, it retrospectively observed the prescription practices for amitriptyline monotherapy—frequent both in prescription count and dosing pattern—and adjunctive therapy in which aripiprazole was added to an antidepressant, as well as how the dosages of these agents related to treatment continuation, expressed as the hazard ratio for discontinuation. Treatment continuation serves as a pragmatic real-world proxy for the overall balance between efficacy (symptom relief) and tolerability, which is a critical clinical goal in the long-term management of chronic conditions like MUOS. For amitriptyline, a dosage of around 25 mg/day was linked to a relatively low hazard of discontinuation. In the group receiving adjunctive therapy with aripiprazole, the analysis suggested that a maximum dose of 1.7–1.8 mg/day was associated with a relatively lower hazard of dropout. However, these findings represent statistical associations from observational data and must be interpreted with caution, particularly in light of potential indication bias.

### 3.1. Clinical Implications of Amitriptyline

By employing a Cox proportional hazards model, we identified a nonlinear relationship between dosage and the risk of discontinuation. In all age groups, risk sharply rose below 5 mg/day, temporarily flattened near 10 mg/day, and then reached a relative minimum around 25 mg/day. Around 30 mg/day, there was a slight uptick, followed by a return to lower levels above 40 mg/day.

As described above, the U-shaped behavior observed in the low-dose range and the valley-shaped behavior seen in the higher-dose range can be interpreted as results supporting the conventional clinical sense that ‘early discontinuation occurs due to limited response in patients with a low maximum tolerable dose’, ‘issues with side effect tolerability at doses around 40 mg/day’, and that the ‘best balance of symptom improvement and tolerability’ is achieved in the intermediate range. The background for tolerating only low doses is thought to include cases where side effects (dry mouth, constipation, dizziness, etc.) precede symptom improvement, making it difficult for patients to continue medication [32]. Furthermore, comparing age-specific curves, the hazard tended to be slightly higher overall in the older age group. This is thought to reflect that risks due to anticholinergic effects, such as easier dehydration, orthostatic hypotension, and cognitive decline, are more likely to manifest in the older group than in the younger group [33,34,35]. As we have previously pointed out, side effects of amitriptyline are particularly problematic in older patients and those with comorbid conditions, becoming a barrier to dosage setting [21]. Since the clinical response to amitriptyline is not necessarily enhanced in a dose-dependent manner, an approach of starting at 5 mg, gradually increasing, and maintaining around 20 mg/day is presumed to be a safer range for older patients, considering side effects and future risks of dementia and falls. This also supports the dosage identified in studies of clinically appropriate dosing for older patients with BMS (ages 65–74) [21].

In a study that divided amitriptyline dosage into three groups for BMS and compared them using VAS, it was reported that the 10–15 mg group was most beneficial for symptom improvement [36]. However, Nagamine’s study used the pain score (VAS) at 4 weeks after treatment initiation as a reference indicator; thus, 10–15 mg, which yields the maximum short-term clinical improvement, was identified as the recommended dose at 4 weeks post prescription. In contrast, our real-world study used a long-term balance of clinical improvement and tolerability, specifically a clinically suitable dosage that includes treatment continuation rates, as an indicator. As a result, the nadir was located around 25 mg, where symptom control is maintained with acceptable side effects. The difference in assessment indicators is thought to have manifested as a difference in dosage.

On the other hand, the phenomenon where the hazard decreased again at 40 mg/day or higher is likely strongly influenced by survivor bias, where some treatment responders who experienced symptom improvement and could tolerate side effects reached the high-dose range and were able to continue treatment. In reality, the number of patients who reached this high-dose range and continued treatment was relatively small, and cautious interpretation is needed regarding the stability of the hazard ratio estimates in this range. Based on these results, high dosages should not be routinely recommended; individualized dosage adjustments while carefully monitoring the side effect profile are essential.

It is also critical to acknowledge that inter-individual variability in response and tolerability to amitriptyline is significant. This variability is partly explained by pharmacogenomic factors, particularly genetic polymorphisms in the cytochrome P450 enzymes responsible for its metabolism, such as CYP2D6 and CYP2C19 [37]. Depending on their genetic makeup, patients can be poor, intermediate, extensive, or ultrarapid metabolizers, which directly impacts plasma concentrations of the drug and its active metabolites, influencing both efficacy and the risk of adverse effects. While our study did not include pharmacogenomic testing, this underlying genetic diversity is a crucial factor in real-world practice and a promising avenue for future research aimed at personalizing MUOS treatment.

Finally, while the average treatment duration observed was approximately nine months, it is important to contextualize this within the chronic nature of MUOS. For many patients, these conditions are debilitating and long-lasting. In the absence of curative therapies, the clinical goal shifts to long-term oral symptom management to maintain quality of life. Therefore, extended treatment may be deemed appropriate based on an individualized risk–benefit assessment, even with medications that carry potential long-term risks (such as those classified as Potentially Inappropriate Medications (PIMs) in older populations), provided that careful monitoring is in place [38].

Previously, symptom improvement with amitriptyline for MUOS has been reported, but a consensus on the clinically appropriate dosage had not been established [11,12,19]. From real-world clinical data, cases where treatment was continued at dosages of 20 mg/day or higher were also observed, but this is based on judgments tailored to individual patient conditions. On the other hand, for patients who can only tolerate low doses around 10 mg/day or who show a poor clinical response, rather than continuing amitriptyline indefinitely, considering other treatment options early, such as the aforementioned aripiprazole adjunctive therapy, may be a rational approach to increase the likelihood of achieving a sustainable balance between oral symptom relief and tolerability.

As shown in Table 6, duloxetine was prescribed in a certain number of cases at our clinic, but this was due to its use as an alternative medication following a supply shortage of amitriptyline around 2023.

### 3.2. Considerations for Adjunctive Aripiprazole Therapy

The group to which aripiprazole was added (hereinafter, the aripiprazole adjunctive group) showed a statistically significantly higher hazard ratio (risk) of study discontinuation, approximately 4.75 times, compared to the group to which it was not added. However, several important points need to be considered when interpreting this result.

First, the observed increase in discontinuation risk in the aripiprazole adjunctive group is likely not a direct reflection of the pharmacological action of aripiprazole itself. In actual clinical practice, adjunctive therapies like aripiprazole tend to be chosen for so-called treatment-resistant cases in which sufficient symptom improvement was not achieved with prior antidepressant monotherapy, or for MUOS patients who present with more severe and complex symptoms. In other words, the aripiprazole adjunctive group likely comprised patients who already had a high risk of treatment discontinuation at study entry (or at the point of aripiprazole initiation), indicating a significant indication bias. Although this study’s model adjusted for age and sex, it may not have fully accounted for other critical confounding factors associated with treatment discontinuation, such as MUOS severity, duration of illness, response to prior treatments, and comorbid psychiatric conditions. Therefore, the overall elevation in hazard ratio must not be misinterpreted as evidence that adjunctive aripiprazole itself impairs treatment continuation.

When the Cox model was stratified by diagnosis (Table 5), adjunctive aripiprazole was linked to a 31% lower hazard of treatment discontinuation in patients diagnosed with OD (HR = 0.69). Although exploratory, this finding is mechanistically plausible: neuroimaging and phenomenological work suggest that OD involves dysfunctional striato-cortical dopaminergic modulation and heightened affective–sensory coupling in the oral somatosensory network [39,40,41,42]. Low-dose aripiprazole’s complex pharmacology may be particularly suited to address this pathophysiology. Its unique profile includes partial agonism at dopamine D2/D3 receptors, which may stabilize aberrant dopaminergic signaling implicated in central pain processing and dysfunctional striato-cortical pathways [43,44]. Concurrently, its potent partial agonism at serotonin 5-HT1A receptors and antagonism at 5-HT2A receptors likely contribute to anxiolytic and mood-stabilizing effects [45]. This dual action on both sensory (dopaminergic) and affective (serotonergic) pathways may effectively address the comorbid mood and anxiety symptoms common in MUOS, thereby enhancing perceived treatment efficacy and supporting adherence to concomitant antidepressants. A recent receptor-oriented review highlights that chronic intra-oral pain conditions such as BMS are characterized by a synergistic interplay between hyperactive NMDA signaling and reduced striatal D2 receptor function. Modulating these axes—via low-dose dopamine-partial agonism coupled with indirect glutamatergic dampening—may, therefore, represent a rational disease-targeted strategy that complements our real-world findings [45,46]. Should this diagnosis-specific advantage be confirmed, early aripiprazole augmentation might be preferentially considered for OD, whereas other MUOS subtypes may require alternative escalation strategies. Prospective subtype-enriched trials integrating patient-reported outcomes with functional imaging will be crucial to verify this targeted benefit and to refine personalized dosing algorithms.

On the other hand, the finding that 1.7–1.8 mg/day was the minimum hazard range in the intra-group analysis of the aripiprazole adjunctive group is noteworthy. Observations from the aripiprazole combination group showed a trend towards a relatively lower treatment discontinuation hazard in the dosage range of 1.7–1.8 mg/day, but this is merely an observed association, suggesting that escalation beyond this dosage may not necessarily contribute to treatment continuation. Indeed, this observed hazard ratio of approximately 0.30 at a dosage of 1.7–1.8 mg/day signifies a substantial potential reduction in the risk of treatment discontinuation by roughly 70% for patients within this specific dosage range of adjunctive aripiprazole therapy. The observed non-sigmoid (U-shaped) dose–response relationships for both amitriptyline and aripiprazole, particularly the decreased treatment continuation at higher doses for aripiprazole, suggest complex underlying pharmacological mechanisms beyond simple receptor saturation. This pattern could be indicative of a narrow therapeutic window where an optimal balance of efficacy and tolerability is achieved, possibly reflecting phenomena such as hormesis, the activation of counter-regulatory mechanisms, or the involvement of multiple binding sites with varying affinities and effects at different concentrations, leading to a diminished benefit or increased side effects at higher dosages [47]. This highlights the critical importance of precise individualized dosage calibration to achieve optimal treatment continuation, rather than pursuing a strategy of uniform dose escalation. This might reflect the action of aripiprazole as a dopamine receptor partial agonist. Notably, this U-shaped dose–response pattern in MUOS differs from the almost monotonically decreasing pattern reported in treatment-resistant depression, where ‘symptom improvement reaches a plateau at 2–5 mg/day’ [48]. Such a pattern suggests that the clinically suitable dosage profile of aripiprazole for MUOS may differ from that for depression. From these results, establishing a clinically appropriate dosage for adjunctive aripiprazole therapy for MUOS is an important future task. Considering the suggestion of a clinically suitable dosage profile different from that for depression, future prospective trials by subtype and dosage are needed to clarify ‘which patients should be introduced to what dosage, when, and how much’.

The predominance of female patients in our sample (86.5% in the amitriptyline group, 87.1% overall) is consistent with the known epidemiology of MUOS conditions like BMS and PIFP. Emerging epidemiological and mechanistic data indicate that the striking female predominance seen in MUOS entities such as Burning Mouth Syndrome and Persistent Idiopathic Facial Pain is at least partly hormonally driven. During the peri- and early post-menopausal transition—when circulating estrogen and progesterone plummet and the estrogen-to-progesterone ratio shifts—neuroactive steroids that normally dampen the trigeminal system excitability are depleted, TRPV1 and NGF expression in oral–facial tissues is up-regulated, and small-fiber integrity may be compromised [49]. These endocrine-mediated changes converge precisely on the age window (mid-50s) in which cohort studies report the highest incidence of both conditions, supporting a causal link between sex hormone decline and disease onset. Furthermore, there may be sex-based differences in central pain processing mechanisms, healthcare-seeking behavior, and the prevalence of comorbid affective conditions such as anxiety and depression [50,51,52]. Therefore, our sample is considered representative of the patient population typically encountered in clinical practice for MUOS.

### 3.3. Study Limitations and Future Perspectives

This study has several important limitations that must be acknowledged.

1. Descriptive and Observational Nature: This study is descriptive and retrospective. Therefore, it can only establish statistical associations derived from statistical models and cannot infer causality. The findings are hypothesis-generating and require validation through prospective randomized controlled trials (RCTs) to establish definitive treatment strategies.
2. Generalizability: Due to the single-center design based in a specialized psychosomatic dentistry clinic, treatment policies and patient background diversity may not have been sufficiently adjusted, and the findings may not be generalizable to primary care or general psychiatric settings.
3. Residual Confounding and Indication Bias: Critical clinical variables such as symptom intensity (e.g., CGI, Patient Global Impression of Change (PGIC)) and quality of life were not systematically collected for this analysis. While we conducted a preliminary analysis incorporating the initial pain VAS score, it did not emerge as a significant predictor and was excluded from the final model to avoid overfitting. The absence of comprehensive severity data means significant residual confounding likely remains. In particular, indication bias is a major concern in the aripiprazole adjunctive group, as this treatment was likely reserved for more severe or treatment-resistant cases.
4. Exclusion of Psychiatric Comorbidities: Our exclusion criteria removed patients actively treated for major psychiatric conditions in other departments. This created a selection bias, meaning our sample may not represent the full spectrum of MUOS patients and precluded the appropriate analysis of psychiatric comorbidities as a covariate in the remaining population.
5. Lack of Intrinsic Patient Parameters: As a retrospective chart review, we were limited to data available in medical records. We lacked systematic data on intrinsic factors such as pharmacogenomic profiles (e.g., CYP2D6/CYP2C19 polymorphisms), detailed psychological assessments, or biomarkers.
6. Geriatric Factors: Given the average age of the cohort (around 60 years), specific geriatric factors such as frailty scores, comprehensive comorbidity indices, and detailed polypharmacy data were not systematically available and, thus, could not be adjusted for in the analysis.
7. Aripiprazole was characterized using the ‘maximum dosage used’, but actual dosage fluctuations are possible.
8. Another limitation is that the detailed profile of side effects and their subjective impact on patients were not sufficiently examined.
9. Since the prescribed dosages of medications are determined by the judgment of each dentist, discretionary differences among attending dentists may be included.
10. Interpretation of Findings: This study is an investigation of actual conditions and does not appraise the appropriateness or safety of individual treatment choices. The identified dosage ranges are observational associations and must not be interpreted as prescriptive recommendations. All treatment decisions require a careful individualized risk–benefit assessment based on the patient’s clinical profile and needs.

Nevertheless, the significance of being able to present clinically relevant dosage guidelines of approximately 25 mg/day for amitriptyline and 1.7–1.8 mg/day for aripiprazole from real-world data is substantial. In MUOS, treatment continuation rates tend to be low, so achieving a sustainable balance between symptom relief and side effects through careful dosage adjustment is paramount to the overall treatment outcome. Future prospective RCTs are essential. These studies should incorporate standardized regimen initiation timing and dosage adjustments, and examine combinations of patient-reported outcomes (PROs), pain-related QOL, and central nervous system indicators such as Single Photon Emission Computed Tomography (SPECT), to establish robust, evidence-based, and personalized treatment strategies for MUOS.

## 4. Materials and Methods

**Figure 3:**
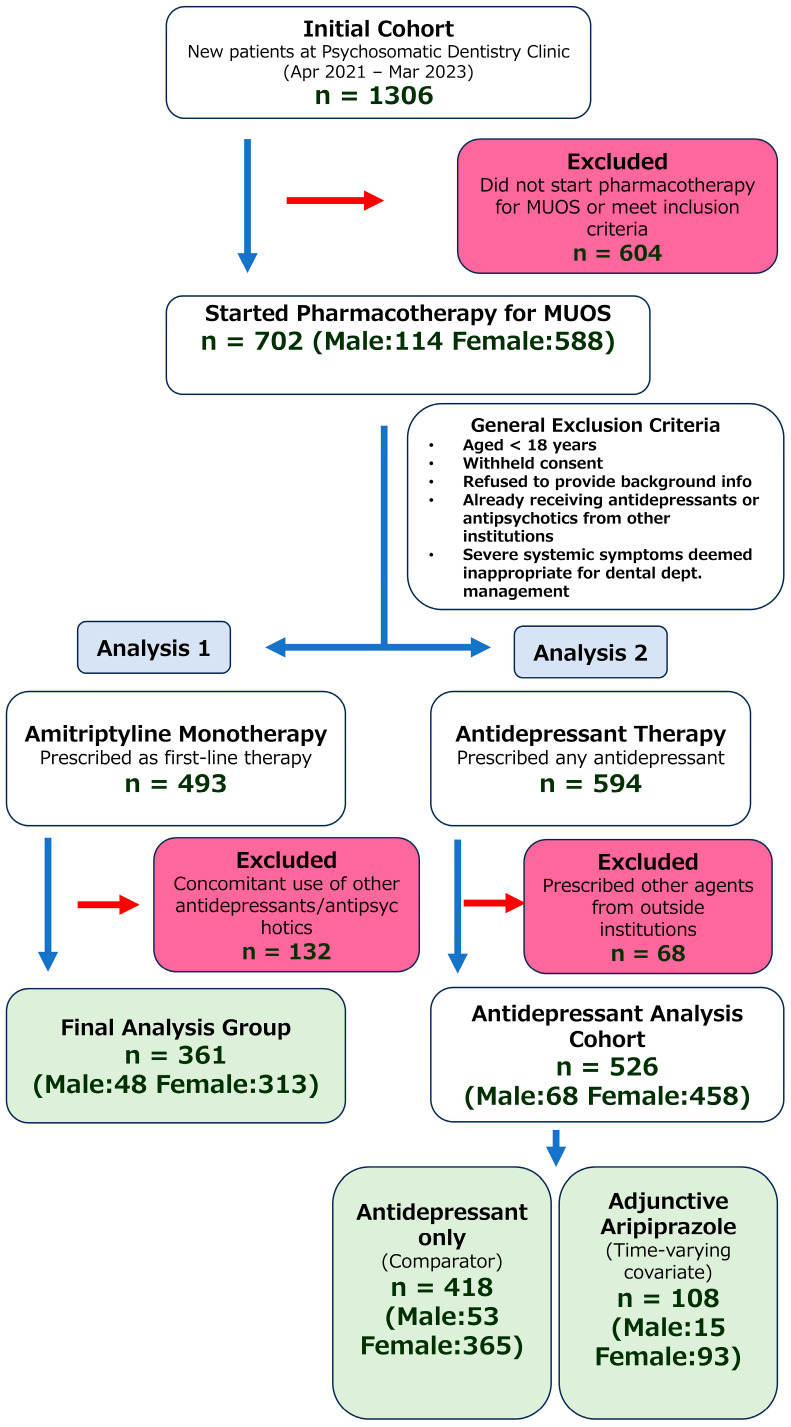
Flowchart of patient selection for the study. The diagram illustrates the process of identifying eligible patients from the initial cohort of individuals who visited the clinic, applying exclusion criteria, and separating them into the analytical groups for amitriptyline monotherapy and antidepressant–aripiprazole adjunctive therapy. MUOS: Medically unexplained oral symptoms/syndromes.

This retrospective investigation included patients who first presented to the Psychosomatic Dentistry Clinic at Tokyo Medical and Dental University Hospital for their initial visit between April 2021 and March 2023 and subsequently began pharmacotherapy for MUOS. Of the total 1306 patients, 702 (53.8%, 702/1306) began pharmacotherapy at our clinic for MUOS. Figure 3 presents a flowchart of the patient selection process.

### 4.1. Diagnostic Ascertainment and Coding (ICD-11 and DSM-5-TR)

All patients underwent a standardized dental and medical assessment to exclude local pathology (mucosal disease, dental/occlusal causes, neuropathies, and structural lesions). Diagnostic labels were assigned using a two-layer framework: (i) ICD-11 disease entities where available; and (ii) a psychiatric trans-diagnostic specifier (DSM-5-TR SSD or ICD-11 BDD) when cognitive-affective features were prominent.

- BMS—coded ICD-11 DA0F.0—required chronic intraoral burning or dysesthetic pain occurring on ≥50% of days for >3 months, absence of mucosal lesions or other explanatory disease on examination/investigation, and clinically meaningful distress or functional interference.
- PIFP—mapped in ICD-11 to 8B82.1 (atypical facial pain)—was characterized by daily poorly localized facial/oral pain of dull/aching quality for >3 months, normal neurological examination and imaging, and no dental cause.
- Oral cenesthopathy—persistent abnormal oral sensations (e.g., foreign-body, crawling, excessive saliva) without objective findings—was diagnosed clinically by experienced dentists. Given the absence of a stand-alone ICD-11 disease code, cases with disproportionate health anxiety or preoccupation were additionally described under ICD-11 BDD and/or DSM-5-TR SSD; where fixed non-bizarre somatic delusions dominated, ICD-11 delusional disorder (6A24) was considered.
- PBS—a persistent conviction of “wrong bite” without verifiable occlusal discrepancy—was diagnosed clinically with corroboration from prior guidelines. As with oral cenesthopathy, PBS may be described dimensionally under BDD (ICD-11) or SSD (DSM-5-TR) when excessive symptom-focused cognitions/behaviors are present.

In keeping with the dental perspective of MUOS, we report outcomes by disorder-specific labels (BMS, PIFP, OC, PBS). In parallel, we indicate when cases also fulfilled SSD (DSM-5-TR) or BDD (ICD-11) to provide cross-walks for readers from psychiatry and general medicine.

Table 6 shows the first medication prescribed at our department. A total of 594 patients (84.6%, 594/702) received some form of antidepressant. Among these, 526 (74.93%, 526/702) were not being treated with other antidepressants or antipsychotics from outside institutions. Amitriptyline was prescribed as the first choice in 493 patients (70.2%, 493/702), of whom 361 (51.4%, 361/702) had no prior prescription of antidepressants or antipsychotics from other institutions. Regarding the use of DPAs, 76 patients received it as the initial prescription in our department (75 of these were aripiprazole), and 108 patients (20.53%, 108/702) had aripiprazole added in combination with some form of antidepressant prescribed at our department.

The reasons for restricting the analysis to the specified medications are as follows: Amitriptyline is the most frequently prescribed drug in our department and is representative of monotherapy that yields sufficient statistical power. While other medications like carbamazepine, valproic acid, and diazepam were prescribed in a small number of cases, these agents, though known primarily as antiepileptics or anxiolytics, are also frequently used off-label for the management of neuropathic pain (a presumed component of conditions like PIFP and BMS) to modulate neuronal hyperexcitability. Meanwhile, other medications like duloxetine had limited prescription numbers, and their improvement of symptoms was also insufficient. Hence, they were excluded as they did not align with the practical objective of this study, which is ‘to support decision-making in clinical settings’. Regarding aripiprazole, clinical improvement in monotherapy has been limited. Moreover, real-world prescribing largely centers on adjunctive therapy, with 108 adjunctive cases versus 75 cases of monotherapy. From a statistical power perspective, focusing on adjunctive therapy for aripiprazole was judged more appropriate.

### 4.2. Human Ethics and Consent to Participate Declarations

All participants provided written informed consent prior to study enrollment. The consent form explicitly stated that de-identified clinical information—including diagnoses, medication choices, dosing and schedule, dose modifications, concomitant therapies, and temporal patterns of care—would be collected and analyzed for research purposes and publication. Accordingly, patients were made aware that their treatment patterns would be analyzed as part of this study. The study protocol was reviewed and approved by the Tokyo Medical and Dental University Hospital Ethical Committee (S2024-011), and the research was conducted in accordance with the principles of the Declaration of Helsinki. This study is an observational descriptive report of routine clinical practice, not an interventional trial. Given the lack of established guidelines or specifically approved medications for MUOS, clinicians relied on treatments used for analogous conditions. No study-driven dose adjustments or treatment allocations were implemented; all prescriptions were determined by the attending physician’s clinical judgment in the patient’s best interest.

### 4.3. Exclusion Criteria

The following patients were excluded: (i) aged < 18 years; (ii) those who withheld consent; (iii) those who refused to provide most of the requested social background information; (iv) those already receiving prescriptions for antipsychotics or antidepressants in psychiatry or related departments; and (v) those who exhibited severe systemic symptoms such that management within the dental department was deemed inappropriate.

### 4.4. Basic Prescribing Policies

For amitriptyline, the initial dosage was set at 5 mg/day or 10 mg/day in consideration of factors such as age, medical history, and concomitant medications, followed by incremental adjustments. Dosage was then modified while carefully monitoring side effects, and, at the time when (1) symptom improvement was deemed sufficient, (2) side effects remained within acceptable limits, or (3) further increases in dosage would only exacerbate side effects without additional symptom improvement, the dosage at that point was documented as the maintenance level for that patient and used for analysis.

Regarding aripiprazole, administration started at 0.5 mg/day or 1 mg/day, with subsequent adjustments following the same approach. However, based on our practical experience that doses exceeding 3 mg/day show limited additional benefit, the maximum dose in this study was set at 3 mg/day [18]. For patients receiving aripiprazole, we recorded the maximum dosage used during the treatment period.

**Figure 4:**
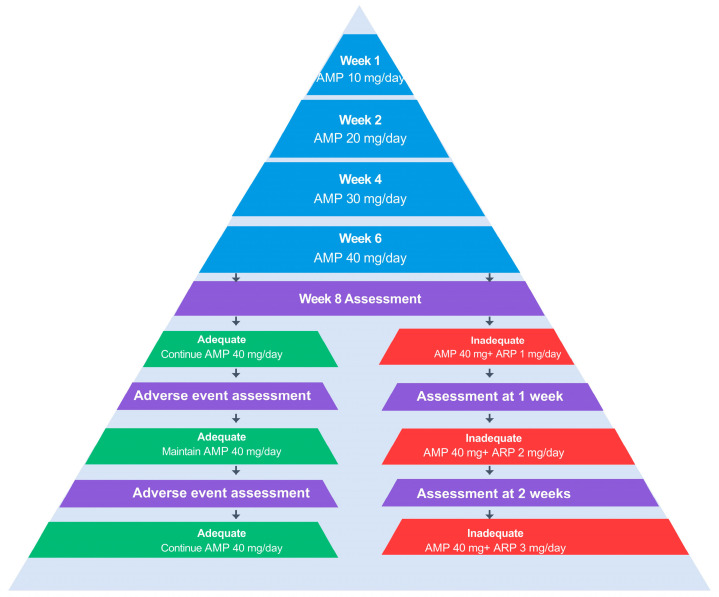
Step-wise titration and augmentation protocol for amitriptyline. The flow chart depicts dose escalation from 10 mg/day in week 1 to 40 mg/day by week 6, followed by weekly efficacy and safety assessments. If the week 8 assessment shows an inadequate response, aripiprazole 1 mg/day is added; this adjunct dose can be increased to 2 mg/day (after the 1-week assessment) and 3 mg/day (after the 2-week assessment) as needed. Patients who achieve an adequate response at any assessment remain on amitriptyline 40 mg/day with or without the current aripiprazole dose. The protocol is intended as a clinical guide; final dosing and timing should be individualized, and adverse effects should be monitored throughout titration. AMP: amitriptyline, ARP: aripiprazole.

Figure 4 depicts the step-wise titration regimen that is most frequently used in our department. In routine practice, however, the actual prescription must be tailored to each patient after a comprehensive appraisal of age, medical history, and the expected benefits and potential adverse effects of amitriptyline and/or aripiprazole. By gradually up-titrating amitriptyline in this manner, we can minimize early adverse effects while giving patients adequate time to adapt to them.

### 4.5. Study Variables

Data on patient age, sex, the diagnosis made at our department (multiple diagnoses possible), the start date of antidepressant prescription at our department, the aripiprazole add-on date, the final observation date, and presence or absence of side effects were extracted from electronic medical records.

### 4.6. Definition of Dropout

In this study, dropout was defined as ‘the point at which pharmacotherapy was discontinued’. Specifically, it refers to situations in which the attending dentist intended to continue, but the patient discontinued medication for some reason. Cases in which patients dropped out before being prescribed medication after the first visit were not included in the observation. Additionally, unavoidable switches to alternative medications due to unstable drug supply or the initiation of new prescriptions at other institutions were also considered ‘dropout’ because they would prevent the analysis of the continuation specific to the medication in question.

### 4.7. Examination of Amitriptyline Monotherapy

To explore factors related to the risk of discontinuing amitriptyline, we employed a multivariable Cox proportional hazards model (Table 3 and Table 7). The outcome variable (time) was ‘the number of days from the start of amitriptyline prescription to discontinuation or the final observation date’, the event was ‘prescription discontinuation’, and censoring was ‘continuation’. Age (modeled as a linear term), sex, the diagnosis made at our department, and the observed maintenance dosage (mg/day) of amitriptyline for each patient were included as explanatory variables. The dosage was handled with a 3-knot B-spline (knots at the 25th, 50th, and 75th percentiles) to allow for nonlinearity. Analyses were conducted using Python and the lifelines package, and adjusted hazard ratios were calculated for different age points (40, 50, 60, and 70 years) for each dosage.

### 4.8. Examination of Adjunctive Therapy Combining Antidepressants with Aripiprazole

In this part, we constructed a Cox proportional hazards model with “aripiprazole add-on” as a time-varying covariate, targeting any antidepressant (including amitriptyline) prescribed (Table 5 and Table 8). Each participant’s observation was divided into two intervals: “period without aripiprazole use” and “period after starting aripiprazole.” Variables such as aripiprazole use (yes/no), age, sex, the diagnosis made at our department, and maximum aripiprazole dosage were entered as explanatory variables. In line with the amitriptyline model, maximum aripiprazole usage was modeled as a continuous variable with a 3-knot B-spline, allowing for nonlinearity. Regression coefficients, hazard ratios (HR), and 95% confidence intervals were computed.

### 4.9. Statistical Analysis

Statistical analyses were conducted using Python (version 3.10.12) and IBM SPSS Statistics (version 29.0; IBM Corporation, Armonk, NY, USA). Continuous variables are presented as mean ± Standard Deviation (SD). To analyze the time to treatment discontinuation, multivariable Cox proportional hazards models were employed. The nonlinear relationship between medication dosage and discontinuation risk was modeled using B-splines. The significance of individual coefficients was assessed using the Wald test, and the overall significance of the spline terms (e.g., for assessing nonlinearity) was evaluated using the Wald omnibus test. The overall model significance was assessed using the log likelihood ratio test. A two-sided *p* < 0.05 was considered statistically significant for all analyses.

## 5. Conclusions

This study descriptively showed the prescription of antidepressants and DPAs to MUOS patients in real-world clinical practice and demonstrated the association between prescription practices during amitriptyline monotherapy and adjunctive aripiprazole therapy and treatment continuation. As a result, a trend towards a relatively lower risk of treatment discontinuation was observed at dosages around 25 mg/day for amitriptyline and at a maximum dosage used of around 1.7–1.8 mg/day within the group of patients receiving aripiprazole combination therapy. These findings describe one aspect of the actual state of pharmacotherapy for MUOS in a specialized setting and absolutely do not constitute recommendations for specific treatment methods or dosages. Instead, they highlight that achieving long-term treatment adherence—a proxy for a favorable balance between efficacy and tolerability—is a critical goal in managing these chronic conditions, and emphasize that individualized careful dose titration based on a thorough risk–benefit assessment is essential.

## Figures and Tables

**Table 1:** Conditions encompassed within the category of medically unexplained oral symptoms/syndromes (MUOS).

Conditions	Descriptions
Oral Dysesthesia (OD)	A historical term encompassing BMS and oral cenesthopathy.
Burning Mouth Syndrome (BMS)	BMS is a chronic oral pain disorder characterized by a persistent burning or scalding sensation of the tongue and/or other oral mucosa in the absence of visible mucosal lesions or laboratory abnormalities. It predominantly occurs in peri- and post-menopausal women and is frequently accompanied by dysgeusia (altered taste) and subjective xerostomia despite normal salivary flow. The burning pain typically worsens as the day progresses.
Oral Cenesthopathy	Oral cenesthopathy is characterized by persistent yet clinically unexplained intra-oral sensations—such as a foreign-body sensation, crawling feelings, or excessive salivation—despite normal dental, mucosal, and radiologic findings. The disorder most commonly affects middle-aged to elderly adults, shows a slight female predominance, and often co-occurs with depressive or anxiety disorders, leading to repeated dental consultations and unnecessary procedures. Symptomatic improvement with certain antidepressants or atypical antipsychotics supports the notion of central somatosensory dysregulation rather than peripheral pathology.
Persistent Idiopathic Facial Pain (PIFP)	PIFP is defined as daily facial or dental pain that is poorly localized and lacks any demonstrable dental, neurological, or radiographic cause. The pain is usually deep, dull, or burning, does not respect trigeminal nerve dermatomes, and sensory examination as well as neuroimaging are normal. PIFP predominates in middle-aged women and is frequently accompanied by psychosocial comorbidities such as anxiety, depression, or sleep disturbance.
Phantom Bite Syndrome (PBS)	PBS is characterized by a persistent distressing perception of malocclusion or an “incorrect bite” in the absence of any demonstrable occlusal discrepancy on clinical or radiographic examination. Patients—predominantly middle-aged women—commonly report continuous or fluctuating occlusal discomfort that is often triggered or exacerbated by minor dental interventions, and the condition frequently co-exists with psychiatric comorbidities such as anxiety, depression, or somatoform disorders. Neuroimaging and neurophysiological studies implicate aberrant central sensorimotor processing, suggesting that maladaptive neuroplasticity rather than peripheral dental pathology underlies the syndrome.

ICD-11 codes: BMS DA0F.0; PIFP (atypical facial pain) 8B82.1. OC and PBS currently lack stand-alone ICD-11 entries; when disproportionate symptom-related cognitions/behaviors were present, cases were described under BDD (6C20). Fixed somatic delusional conviction supported delusional disorder (6A24). From a DSM-5-TR perspective, MUOS presentations may fulfill SSD when criteria are met (≥1 distressing somatic symptom plus disproportionate thoughts/feelings/behaviors).

**Table 2:** Baseline demographic and diagnostic characteristics of the 361 patients with medically unexplained oral symptoms/syndromes (MUOS) who initiated amitriptyline monotherapy and were eligible for the treatment continuation (time-to-discontinuation) analysis. Values are number of patients or mean ± SD (Standard Deviation), as appropriate. OD = oral dysesthesia; BMS = Burning Mouth Syndrome; OC = oral cenesthopathy; PIFP = Persistent Idiopathic Facial Pain; PBS = Phantom Bite Syndrome.

Characteristic	Value
Total patients analyzed	361
Sex (male, female)	48, 313
Mean age (mean ± SD), years	60.7 ± 14.5
― Male	56.8 ± 15.6
― Female	61.2 ± 14.2
Clinical diagnoses (multiple diagnoses permitted)	
― OD (BMS + OC)	294
― BMS	254
― OC	74
― PIFP	70
― PBS	17
Mean treatment duration (mean ± SD), days	311.7 ± 341.3

**Table 3:** Multivariable Cox proportional hazards model identifying predictors of amitriptyline discontinuation in the MUOS cohort (n = 361). The table lists log–hazard coefficients (coef), hazard ratios (exp [coef]), standard errors, 95% confidence intervals, Wald z-statistics, and *p*-values. B-spline terms model the nonlinear effect of maintenance dose.

	Coef	Exp (Coef)	Se (Coef)	Coef Lower 95%	Coef Upper 95%	Exp (Coef) Lower 95%	Exp (Coef) Upper 95%	z	*p*-Value
Dose Spline 0	−1.230	0.290	0.860	−2.920	0.470	0.050	1.590	−1.420	0.160
Dose Spline 1	0.560	0.520	0.750	−2.130	0.800	0.120	2.230	−0.890	0.370
Dose Spline 2	−2.120	0.120	0.300	−2.680	−1.510	0.070	0.220	−7.060	<0.005 *
Dose Spline 3	1.280	0.280	0.710	−2.680	0.110	0.070	1.120	−1.800	0.070
Dose Spline 4	−4.020	0.020	1.680	−7.320	0.720	0.000	2.060	−2.390	0.02 *
Dose Spline 5	1.910	0.150	1.010	−3.880	0.060	0.020	1.060	−1.900	0.060
age	0.010	1.010	0.000	0.000	0.010	1.000	1.010	1.190	0.110
sex_F	0.490	1.620	0.180	0.130	0.840	1.140	2.320	2.690	0.01 *
OD	−0.020	0.980	0.240	−0.480	0.450	0.620	1.570	−0.070	0.950
PIFP	0.320	1.370	0.240	−0.150	0.790	0.860	2.190	1.330	0.180
PBS	0.170	1.180	0.280	−0.380	0.710	0.690	2.040	0.610	0.540

* indicates statistical significance. OD: oral dysesthesia, PIFP: Persistent Idiopathic Facial Pain, PBS: Phantom Bite Syndrome.

**Table 4:** Baseline demographic and clinical characteristics of the antidepressant analysis cohort stratified by adjunctive aripiprazole use. This table outlines the characteristics of all patients who initiated antidepressant therapy (n = 526). It provides a comparative breakdown of demographics, clinical diagnoses, and treatment durations for patients who received antidepressant monotherapy (n = 418) versus those who received adjunctive aripiprazole (n = 108).

Characteristic	Value
Total patients analyzed	526
Sex (male, female)	68, 458
Mean age (mean ± SD), years	61.1 ± 14.4
― Male	56.3 ± 16.3
― Female	61.8 ± 13.9
Clinical diagnoses (multiple diagnoses permitted)	
― OD (BMS + OC)	436
― BMS	375
― OC	113
― PIFP	105
― PBS	26
Mean treatment duration for antidepressant-only cases (n = 418), days	366.1 ± 347.9
Adjunctive therapy subgroup (antidepressant + aripiprazole, n = 108)	
― Mean duration of the antidepressant-only period before aripiprazole add-on, days	155.3 ± 121.7
― Mean total treatment duration, days	295.4 ± 315.7
― Mean maximum dose of aripiprazole during treatment, mg	1.3 ± 0.7

SD: Standard Deviation, OD: oral dysesthesia, BMS: Burning Mouth Syndrome, OC: oral cenesthopathy, PIFP: Persistent Idiopathic Facial Pain, PBS: Phantom Bite Syndrome.

**Table 5:** Multivariable Cox proportional hazards model evaluating factors associated with time to discontinuation of antidepressant therapy when adjunctive aripiprazole is treated as a time-varying covariate (total n = 526). The table lists log–hazard coefficients (coef), hazard ratios (exp [coef]), standard errors, 95% confidence intervals, Wald z-statistics, and *p*-values. The model includes age, sex, MUOS subtype, and a B-spline representation of the maximum aripiprazole dose.

	Coef	Exp (Coef)	Se (Coef)	Coef Lower 95%	Coef Upper 95%	Exp (Coef) Lower 95%	Exp (Coef) Upper 95%	z	*p*-Value
age	0.014	1.014	0.004	0.006	0.021	1.006	1.021	3.716	<0.0005 *
sex_F	−0.261	0.771	0.142	−0.540	0.018	0.583	1.019	−1.830	0.067
aripiprazole_added	1.558	4.751	0.199	1.168	1.949	3.216	7.021	7.824	<0.0005 *
OD	−0.371	0.690	0.163	−0.690	−0.051	0.501	0.950	−2.273	0.023 *
PIFP	−0.173	0.841	0.148	−0.464	0.118	0.629	1.125	−1.168	0.243
PBS	0.016	1.016	0.228	−0.430	0.462	0.650	1.587	0.070	0.944
Dose Spline 1	0.415	1.515	0.280	−0.133	0.964	0.875	2.623	1.484	0.138
Dose Spline 2	−0.942	0.390	0.497	−1.915	0.032	0.147	1.032	−1.896	0.058
Dose Spline 3	−1.089	0.337	0.596	−2.257	0.079	0.105	1.082	−1.827	0.068
Dose Spline 4	−0.206	0.814	0.396	−0.982	0.570	0.375	1.768	−0.521	0.603

* indicates statistical significance. OD: oral dysesthesia, PIFP: Persistent Idiopathic Facial Pain, PBS: Phantom Bite Syndrome.

**Table 6:** Statistics of initial prescribed medications in our clinic.

Generic Drug Name	Value
Amitriptyline	493
Imipramine	24
Duloxetine	66
Mirtazapine	9
Setiptiline	1
Fluvoxamine	1
Aripiprazole	75
Sulpiride	1
Pregabalin	7
Mirogabalin	2
Carbamazepine	15
Valproic acid	3
Diazepam	2
Ethyl loflazepate	1
Cepharanthine	2
Total	702

**Table 7:** PECO for the analysis of amitriptyline monotherapy.

P (Population)	New adult patients (aged ≥ 18 years) with medically unexplained oral symptoms (MUOS) who initiated amitriptyline monotherapy at the Psychosomatic Dentistry Clinic at Tokyo Medical and Dental University Hospital between April 2021 and March 2023.
E (Exposure)	Receiving specific observed maintenance dosages of amitriptyline (e.g., 25 mg/day).
C (Comparator)	Receiving other observed maintenance dosages of amitriptyline (e.g., dosages lower or higher than 25 mg/day).
O (Outcome)	Duration of treatment continuation and hazard ratio of treatment discontinuation.

**Table 8:** PECO for the analysis of antidepressant–aripiprazole adjunctive therapy.

P (Population)	New adult patients (aged ≥ 18 years) with medically unexplained oral symptoms (MUOS) who initiated antidepressant pharmacotherapy (including amitriptyline) at the Psychosomatic Dentistry Clinic at Tokyo Medical and Dental University Hospital between April 2021 and March 2023.
E (Exposure)	Receiving antidepressant therapy augmented with aripiprazole (specifically, considering various maximum aripiprazole dosages, e.g., 1.7–1.8 mg/day).
C (Comparator)	Receiving antidepressant therapy without aripiprazole augmentation.
O (Outcome)	Duration of treatment continuation and hazard ratio of treatment discontinuation.

## Data Availability

The datasets generated and analyzed during the current study are not publicly available due to privacy and confidentiality agreements but are available from the corresponding author on reasonable request.

## Abbreviations

The following abbreviations are used in this manuscript:

AMP	Amitriptyline
AO	Atypical Odontalgia
ARP	Aripiprazole
BMS	Burning Mouth Syndrome
CAS	Complex Adaptive System
DPA	Dopamine Receptor Partial Agonists
DSM-5-TR	Diagnostic and Statistical Manual of Mental Disorders, 5th ed., Text Revision
FFR	Full Functional Recovery
HR	Hazard Ratio
ICD-11	International Classification of Diseases 11th Revision
MUOS	Medically Unexplained Oral Symptoms/Syndromes
OD	Oral Dysesthesia
PBS	Phantom Bite Syndrome
PGIC	Patient Global Impression of Change
PIFP	Persistent Idiopathic Facial Pain
PIMs	Potentially Inappropriate Medications
PROs	Patient-Reported Outcomes
RCTs	Randomized Controlled Trials
SD	Standard Deviation
SNRI	Serotonin-Noradrenaline Reuptake Inhibitors
SPECT	Single Photon Emission Computed Tomography
SSRI	Selective Serotonin Reuptake Inhibitors
TCA	Tricyclic Antidepressants
